# Androgen receptor increases hematogenous metastasis yet decreases lymphatic metastasis of renal cell carcinoma

**DOI:** 10.1038/s41467-017-00701-6

**Published:** 2017-10-13

**Authors:** Qingbo Huang, Yin Sun, Xin Ma, Yu Gao, Xintao Li, Yuanjie Niu, Xu Zhang, Chawnshang Chang

**Affiliations:** 1 Department of Urology, Chinese PLA General Hospital, Beijing, 100853 China; 2 George Whipple Lab for Cancer Research, Departments of Pathology, Urology, Radiation Oncology, and The Wilmot Cancer Center, University of Rochester Medical Center, Rochester, NY 14642 USA; 3 Chawnshang Chang Sex Hormone Research Center, Tianjin Institute of Urology, Tianjin Medical University, Tianjin, 300211 China; 4 Sex Hormone Research Center, China Medical University/Hospital, Taichung, 404 Taiwan

## Abstract

Clear cell renal cell carcinoma (ccRCC) is a gender-biased tumor. Here we report that there is also a gender difference between pulmonary metastasis and lymph node metastasis showing that the androgen receptor (AR)-positive ccRCC may prefer to metastasize to lung rather than to lymph nodes. A higher AR expression increases ccRCC hematogenous metastasis yet decreases ccRCC lymphatic metastases. Mechanism dissection indicates that AR enhances miR-185-5p expression via binding to the androgen response elements located on the promoter of miR-185-5p, which suppresses VEGF-C expression via binding to its 3′ UTR. In contrast, AR-enhanced miR-185-5p also promotes HIF2α/VEGF-A expression via binding to the promoter region of HIF2α. Together, these results provide a unique mechanism by which AR can either increase or decrease ccRCC metastasis at different sites and may help us to develop combined therapies using anti-AR and anti-VEGF-C compounds to better suppress ccRCC progression.

## Abstract (web summary)

The incidence of renal cell carcinoma is higher in males than in females due to the different androgen receptor signaling but the molecular mechanisms behind this gender bias are unclear. Here the authors show how androgen receptor expression influences the metastatic route through the regulation of miR-185 and VEGF isoforms.

## Introduction

Renal cell carcinoma (RCC) is the most lethal of all urological malignancies^1^, accounting for 2–3% of adult malignancies. Approximately 30% of RCC patients had metastatic lesions detected at initial diagnosis^2^, however, the detailed mechanisms behind the development of these metastases remain unclear. The most common histologic subtype, clear cell RCC (ccRCC), derives from the epithelial cells of the proximal renal tubule and may be resistant to many chemotherapies and radiotherapies. Early studies indicated that the majority of ccRCC were associated with a loss of the von Hippel–Lindau (VHL) gene with induction of hypoxia-inducible factors (HIF) and vascular endothelium growth factors (VEGF)^3, 4^, and anti-VEGF targeted therapy remains the first-line treatment for metastatic ccRCC.

The ccRCC is a gender-biased neoplasm^5, 6^ that involves the alterations of the androgen/androgen receptor (AR) signals^7–9^. Early studies indicated that the AR acted as a stimulator to promote the ccRCC progression^7^. However, some clinical surveys also suggested that AR expression was associated with less malignancy^8^ and AR-negative tumors might be associated with more metastatic ccRCC^9^ with 10 of 16 cases of lymph node metastatic RCC vs. one case of lung metastatic ccRCC^9^. This contrasting difference for the AR roles in ccRCC progression suggests that the AR may play distinct roles in a stage- or tissue-specific manner. The detailed mechanisms, however, remain unclear.

Previous studies indicated that hematogenous metastasis and lymph node metastasis of RCC were characterized with varying microvessel density (MVD) and angiogenesis-specific factors^10^. Interestingly, from a survey of about 119 ccRCC specimens, we found increased AR expression in the pulmonary metastases (PM), the most common hematogenous metastatic site, yet decreased AR expression was linked with metastases to the lymph nodes. These differential manifestations of the two ccRCC metastatic sites suggested that the role of AR in hematogenous vs. lymphatic ccRCC metastasis may vary and may function through different mechanisms to modulate different target genes.

Here we investigated the underlying mechanisms behind these clinical findings and found AR might differentially modulate VEGF-A vs. VEGF-C expression via regulation of the microRNA (miRNA), miR-185-5p, to either promote or suppress RCC metastasis to different sites.

## Results

### AR expression in pulmonary vs. lymphatic metastatic ccRCC

**Fig. 1:**
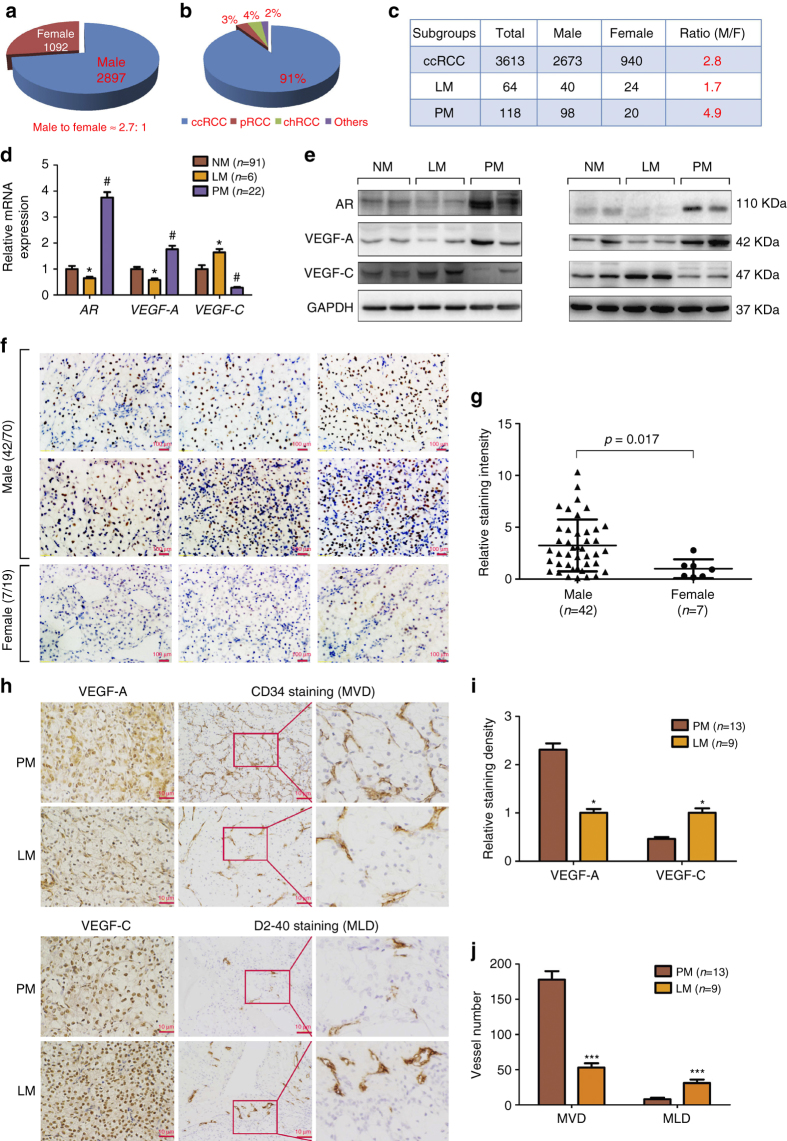
Differential AR expression in pulmonary vs. lymphatic metastasis of ccRCC. **a**–**c** Gender difference of RCC incidence in 3989 cases from the Chinese PLA Hospital. The *left* pie chart (**a**) indicates the constituent ratio of male and female and the *right* pie chart (**b**) indicates the constituent ratio of tumor subtypes in this RCC cohort. The table (**c**) indicates the gender difference in different groups including ccRCC population, pulmonary metastatic (PM) ccRCC, and lymphatic metastatic (LM) ccRCC. **d** mRNA expressions of AR, VEGF-A, and VEGF-C in non-metastatic ccRCC (NM, *n* = 91), LM ccRCC (*n* = 6), and PM ccRCC (*n* = 22) in 119 cases of ccRCC specimens. The AR expression was normalized against TATA box binding protein (TBP) expression.*p < 0.05, LM was compared to NM, and #*p* < 0.05, PM was compared to NM. **e** Protein levels of AR, VEGF-A, and VEGF-C were detected by western blot in NM, LM, and PM specimens. **f** Representative AR staining in various ccRCC tumors showed that AR expression was mainly located in the nucleus. *Scale bar*, 100 μm. **g** The AR expression intensity of AR-positive RCC in males (*n* = 42) was also higher than that in females (*n* = 7) by analyzing the signaling of IHC staining using Image-Pro Plus software. **h** Representative IHC staining of angiogenic and lymphangiogenic microvessel density (MVD (*upper*), and MLD (*lower*), respectively), VEGF-A and VEGF-C in PM (*n* = 13) and LM (*n* = 9). *Scale bar*, 10 μm. **i** Quantitation of VEGF-A (*upper*) and VEGF-C (*lower*) expressions detected by immunohistochemistry using Image-Pro Plus software. **j** An average of five random views of MVD and MLD were calculated in every slide and mean and standard error were shown for every group. *p < 0.05, ***p < 0.001 by *t*-test for two groups or ANOVA for more than two groups

Using the epidemiological survey of 3989 RCC cases from Chinese PLA General Hospital, we found the gender difference with male to female incidence ratio near 2.7:1 (Fig. 1a). This clinical survey also indicated that 91% of these RCC samples are ccRCC (Fig. 1b), with the gender incidence ratio of male to female at 2.8:1 (Fig. 1c). Interestingly, among those, 118 ccRCC patients who developed PM at diagnosis or within 12 months after surgery had a male to female ratio at 4.9:1 (Fig. 1c). Yet among 64 ccRCC patients who developed the lymphatic metastases (LM), the male to female ratio was at 1.7:1 (Fig. 1c). These contrasting differences in gender ratio in metastatic locations of ccRCC suggest that sex hormones and their receptors may play key roles to influence the ccRCC progression.

We then focused on the AR and examined the AR mRNA expression in 119 randomly extracted ccRCC patients (86 males and 33 females). The results in Fig. 1d revealed that AR expression in the primary ccRCC tissues in patients who developed PM was 3.75-fold higher than that in non-metastatic (NM) ccRCC, and in patients who developed LM the AR expression was roughly 50% lower as compared to the NM ccRCC (Fig. 1d). In addition, angiogenesis-associated factor VEGF-A mRNA expression was upregulated in PM 1.76-folds higher than in NM, yet lymphatic angiogenesis-associated VEGF-C mRNA expression was downregulated in PM with a 72% reduction compared to the NM. In contrast, VEGF-C was upregulated in LM at 1.64-folds more than NM, yet VEGF-A was downregulated in LM at 42% of that in NM (Fig. 1d). The western blot analysis also confirmed that AR and VEGF-A was upregulated in PM compared to LM and NM, while VEGF-C was upregulated in LM relative to PM and NM (Fig. 1e).

These results suggest AR may play key roles for the gender difference in the differential metastatic destinations, and VEGF-A and VEGF-C may play a role consistent with their respective function associated with differential metastatic locations.

We next detected the AR protein expression by IHC in 89 randomly extracted cases of AR-positive or AR-negative ccRCC specimens, using prostate tissues as positive controls (Supplementary Fig. 1). For the Table 1, we used the logistic regression analysis to link AR expression data with other previously reported predictive factors (gender, Fuhrman grade, pT stage, and metastatic status)^8, 9^ that are associated with RCC progression. We also used AR expression (positive or negative) as a dependent variable, and we found that the gender, Fuhrman grade, and pulmonary metastatic status were correlated with AR expression showing a higher AR-staining-positive ratio in PM than in NM and LM specimens. Importantly, we found that the risk of AR positivity in PM was 6.7-fold higher than that found in LM and NM specimens (Table 1). These data also indicated there was a gender difference in expression level for AR. AR-positive ratio in males (42/70, 60%) was much higher than that in females (7/19, 36.8%). Furthermore, the AR expression intensity of AR-positive RCC in males (*n* = 42) was also higher than that in females (*n* = 7, Fig. 1f, g). However, among AR-positive RCCs, we found that the possibility of PM from females was similar to that for AR-positive RCC from males (2/7 vs. 8/42, Fisher’s exact test, *p* = 0.62). In addition, among female patients, AR-positive RCC was more likely to metastasize to lung than AR-negative RCC (2/7 vs. 0/12). Therefore, it was likely that AR level rather than gender (or hormone) determines RCC metastasis.

**Table 1:** Association of AR expression with ccRCC specimens’ characteristics analyzed by logistic regression

**Valuables (** ***n*** **)**	**AR positive (** ***n*** **, %)**	**B**	**SE**	***p*** **-value**	**OR (95% CI)**
*Gender*		−1.627	0.680	0.017*	0.197(0.052–0.745)
Male (70)	42 (60)				
Female (19)	7 (36.8)				
*Fuhrman Grade* ^a^				0.003*	
1 (17)	14 (82.4)				
2 (42)	26 (61.9)	−1.789	0.884	0.043	0.167 (0.030–0.945)
3 (23)	7 (30.4)	−3.615	1.080	0.001	0.027 (0.003–0.224)
4 (7)	2 (28.8)	−4.559	1.428	0.001	0.010 (0.001–0.172)
*pT stage* ^*b*^				0.123	
T1 (54)	31 (57.4)				
T2 (9)	4 (44.4)	−1.350	0.878	0.124	0.259 (0.046–1.449)
T3 (23)	12 (52.2)	1.296	0.745	0.082	3.655 (0.848–15.754)
T4 (3)	2 (62.7)	22.420	15921.320	0.999	5.459E9 (0.000)
*Pulmonary metastasis*		1.904	0.842	0.024*	6.711 (1.289–34.929)
No (76)	39 (51.3)				
Yes (13)	10 (76.9)				
*Lymphatic metastasis*		−21.410	15921.320	0.999	0.000 (0.000)
No (80)	47 (58.8)				
Yes (9)	2 (22.2)				
*Constant*		7.174	3980.330	0.999	1304.430

ccRCC clear cell renal cell carcinoma, CI confidence interval, OR odds ratio (male, non-pulmonary, and lymphatic metastasis as reference), SE standard error

*Statistically significant

^a^Grade 1 as reference

^b^T1 as reference

Although only 2 out of 9 patients with ccRCC with LM showed positive AR staining^9^, we failed to find a significant difference of AR-negative ratio between LM vs. NM ccRCC, which might be due to the limited sample size. Furthermore, we found that seven cases of ccRCC with LM were T3 or T4 stage, which indicated that T stage and LM were dependent variables in this cohort^9^.

As ccRCC cells mainly migrate/invade through the inferior vena cava to colonize lung or via migration through lymphatic vessels to invade the lymph nodes, we also stained ccRCC samples with angiogenesis-specific marker CD34^11^ and lymphatic angiogenesis-specific marker D2-40^12^. The results revealed that PM showed a higher VEGF-A expression and microvessel density (MVD) staining, indicative of more angiogenesis (Fig. 1h–j), while LM showed a higher VEGF-C expression and microvessel lymphoangiogenic density (MLD) consistent with enhanced lymphangiogenesis (Fig. 1h–j).

Together, results from Fig. 1a–j and Table 1 suggest that differential expression of AR can be linked with different metastatic destinations of the primary ccRCC tumor cells, and those ccRCC cells with a higher AR expression may prefer to invade to the pulmonary tissues vs. those with a lower AR expression that may prefer to invade into lymph nodes.

### AR differentially regulates expression of VEGF-A and VEGF-C

**Fig. 2:**
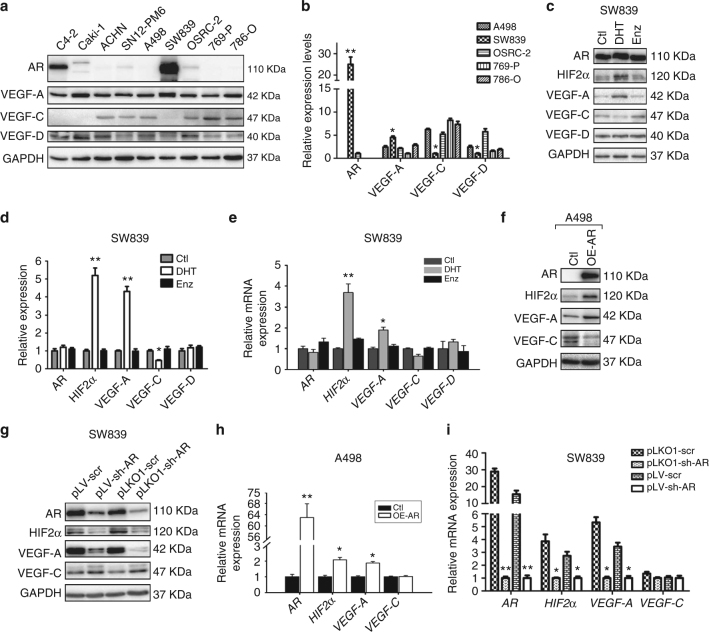
AR differentially regulates the VEGF-A vs. VEGF-C in ccRCC cells. **a** Protein expressions of AR, VEGF-A, VEGF-C, and VEGF-D were detected by western blot in a series of ccRCC cell lines (SN12-PM6, Caki-1, ACHN, A498, SW839, OSRC-2, 769-P, and 786-O). The prostate cancer cell line C4-2 expressing AR was used as positive control. **b** Quantitation of protein expressions of AR, VEGF-A, VEGF-C, and VEGF-D in triplicate western blot assays of VHL mutant cell lines (A498, SW839, OSRC-2, 769-P, and 786-O). **c** Immunoblotting of HIF2α, VEGF-A, VEGF-C, and VEGF-D following 48 h DHT and Enz treatment in SW839 cell line. **d** Quantitation of protein expressions of AR, VEGF-A, VEGF-C, and VEGF-D in triplicate western blot assay. **e** The mRNA levels of HIF2α, VEGF-A, and VEGF-C following 24 h DHT and Enz treatment in SW839 cells. **f** Western blotting of HIF2α, VEGF-A, and VEGF-C in A498 cells with/without AR overexpression (OE-AR). **g** We used two different sh-RNAs (pLKO1-sh-AR and pLV-sh-AR) to knockdown AR in SW839 cells. The results showed that both sh-RNAs could effectively suppress AR. Moreover, HIF2α and VEGF-A were decreased yet VEGF-C was increased. **h**, **i** The mRNA levels of HIF2α, VEGF-A, and VEGF-C in cells with/without AR overexpression in A498 cells (**h**) and AR knockdown in SW839 cells (**i**), TBP was used as an internal loading control. Triplicate experiments were carried out to detect mRNA and protein expressions. *p < 0.05, **p < 0.01, were considered statistically significant by *t*-test

As most ccRCCs (~80–85%) harbor the VHL gene mutation^4, 13, 14^, we examined AR expression in various RCC cell lines including VHL-wild type (VHL-WT) cell lines SN12-PM6, Caki-1, and ACHN and VHL-mutant (VHL-mut) cell lines A498, SW839, OSRC-2, 769-P, and 786-O cells^15–17^. The prostate cancer cell line C4-2 expressing AR was used as positive control (Fig. 2a). Among these cell lines, AR was undetectable by western blot assay in A498 cells, while a much higher AR expression was found in SW839 cells. Furthermore, VEGF-A expression was relatively higher, while VEGF-C expression was relatively lower in SW839 cells than other cell lines. A498 cells expressed average levels of VEGF-A and VEGF-C (Fig. 2a, b). Therefore, these two cell lines were used to further study AR target genes in the VHL-mut ccRCC cells.

We focused on the HIF2α→VEGF-A signal vs. the VEGF-C/VEGF-D signal as the former is strongly correlated with angiogenesis^18, 19^, while the latter is directly linked with lymphangiogenesis^20–22^. These cells were cultured in phenol red-free media with charcoal-stripped serum for 24 h and then treated with the androgen dihydrotestosterone (DHT), vehicle, or the anti-androgen enzalutamide (Enz). The western blot results showed that HIF2α and VEGF-A were increased by DHT, but not by vehicle or Enz in SW839 cells (Fig. 2c, d). However, VEGF-C decreased with DHT treatment, and VEGF-D was almost unchanged (Fig. 2c, d) with all treatments. The quantitative PCR assay confirmed that the mRNA levels of HIF2α and VEGF-A also increased by DHT treatment. However, mRNA levels of VEGF-C and VEGF-D did not show significant differences among these groups (Fig. 2e). Importantly, manipulating AR expression with AR cDNA or AR-short hairpin RNA revealed that adding functional AR in A498 cells increased the expression of HIF2α and VEGF-A with a decrease of VEGF-C expression (Fig. 2f). In contrast, in SW839 cells, knocking down AR with two different AR-shRNAs reduced the expression of HIF2α and VEGF-A, but increased the VEGF-C expression in an AR-level-dependent manner (Fig. 2g).

We also applied the quantitative PCR assay to confirm the above results of mRNA expression and showed that addition of functional AR in A498 cells increased mRNA expression of HIF2α and VEGF-A significantly, while it did not change the mRNA level of VEGF-C (Fig. 2h). Furthermore, knocking down AR with the two sh-ARs in SW839 cells decreased the mRNA levels of HIF2α and VEGF-A dramatically without significantly decreasing the VEGF-C mRNA expression (Fig. 2i). The more effective pLKO1-sh-AR was chosen to perform the other functional assays.

Together, results from Fig. 2a–i indicated that androgen/AR signals could increase HIF2α and VEGF-A at the transcriptional levels while downregulating VEGF-C expression at the post transcriptional levels in the VHL-mut ccRCC cells.

### AR expression influences angiogenesis and lymphangiogenesis

To further confirm the clinical data observed in Fig. 1, we assayed the ccRCC’s capacity to induce angiogenesis using vascular endothelial HUVEC cells (to link to lung metastasis) vs. lymphatic endothelial human dermal lymphatic microvascular endothelial cells (HDLMVEC) cells (to link to lymph node metastasis), as it is generally believed the route of tumor extravasation is influenced by the associated endothelial cells that tumor cells recruit during the metastatic process^23^.

**Fig. 3:**
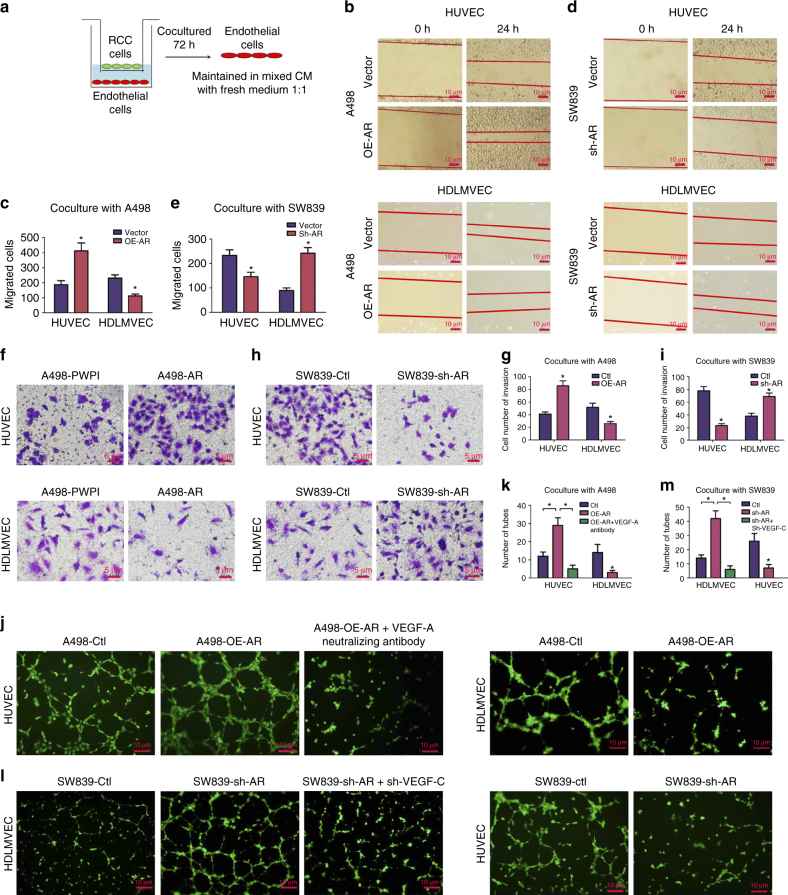
AR of ccRCC cells influences angiogenesis and lymphoangiogenesis. **a** Outline of co-culture system. Endothelial cells (HUVEC and HDLMVEC) were co-cultured with ccRCC cells with indicated treatments (with/without overexpression/knockdown of AR) for 72 h, and then the endothelial cells were harvested. After harvest, the endothelial cells were maintained in mixed conditioned medium (CM from co-culture system) with fresh media at the ratio of 1:1. **b**, **d** Wound-healing assays of HUVEC and HDLMVEC cells co-cultured with A498 **b** and SW839 **d** with altered AR expression at indicated time points. *Scale bar* in **b**, **d** 10 μm. **c**, **e** Quantitation of endothelial cells migration in wound-healing assay after co-culture with indicated ccRCC cells as desc﻿ribed in **b**, **d**. **f**, **h** Transwell invasion assay of HUVEC (*upper*) and HDLMVEC (*lower*) after co-culture with A498 cells overexpressing AR (**f**) and SW839 cells with AR knocked down (**h**). After 24 h, the invaded cells were stained and five random fields per well were analyzed. *Scale bar* in **f**, **h**, 5 μm. **g**, **i** Quantitation of the transwell invasion described in **f**, **h**. **j** A representative image of tube formation assays of HUVEC and HDLMVEC after co-culture with A498 overexpressing AR. The HUVEC cells during coculture with A498 cells were also blocked with VEGF-A neutralizing antibody (R&D Systems) at the concentration of 0.08 µg/ml. The endothelial cells were pre-stained with Calcein AM at the concentration of 2 µM and monitored with fluorescence microscope. **l** Tube formation assay of HUVEC and HDLMVEC after co-culture with SW839 cells with AR knockdown. The SW839 cells were infected with lentivirus for AR and sh-VEGF-C were used for co-culture with HDLMVEC cells for the rescue assay. *Scale bar* in **j**, **l**, 10 μm. **k**, **m** Quantitation of the tube number described in **j**, **l**. The functional assays were all replicated four times. *p < 0.05, was considered statistically significant by *t*-test for two groups and ANOVA for more than two groups

We used the Chamber co-culture system^24^ with endothelial cells co-cultured with ccRCC cells for 72 h followed by harvest of endothelial cells for migration (wound-healing assay), invasion, and tube formation (see cartoon in Fig. 3a). After harvest, the endothelial cells were maintained in mixed conditioned media (CM, from co-culture system) with fresh media at the ratio of 1:1. We found that the CM from A498 cells with added AR could enhance wound healing cell migration in HUVEC cells, yet suppress cell migration in HDLMVEC cells (Fig. 3b, c). Yet in contrast, CM from SW839 cells with knocked down AR could suppress wound healing cell migration in HUVEC cells, yet promote this in HDLMVEC cells (Fig. 3d, e). Invasion assays also showed that adding functional AR into A498 cells resulted in more invasion of HUVEC cells, yet less invasion in HDLMVEC cells (Fig. 3f, g), while knocking down AR in SW839 cells suppressed invasion of HUVEC cells yet promoted the invasion of HDLMVEC cells (Fig. 3h, i).

Together, results from Fig. 3a–i suggest that differential AR expression in ccRCC cells may influence ccRCC cells metastatic destination based upon the differential recruitment of hematogenous endothelial cells vs. lymphatic endothelial cells.

We also applied the tube formation assay^25, 26^ to examine the influences of differential AR expression in ccRCC cells on the induction of the hematogenous angiogenesis vs. lymphatic angiogenesis to confirm the results of Fig. 3a–i. The results revealed that adding AR in A498 cells induced more hematogenous endothelial cell tube formation yet less lymphatic endothelial cell tube formation (Fig. 3j, k). As expected, knocking down AR in SW839 cells induced more lymphatic endothelial cell tube formation while less HUVEC tube formation (Fig. 3l, m).

Importantly, interruption approaches using VEGF-A neutralizing antibody or knocking down VEGF-C in ccRCC cells then partially reversed the AR impacts on the differential effects of in vitro assays of angiogenesis (Fig. 3j–m).

Together, results from Fig. 3 suggest that AR may function through modulation of the VEGF-A or VEGF-C in ccRCC cells to differentially influence the hematogenous and the lymphatic endothelial cells, thus ultimately affect different metastatic destinations.

### AR regulates HIF2α, VEGF-A and VEGF-C via miR-185-5p

**Fig. 4:**
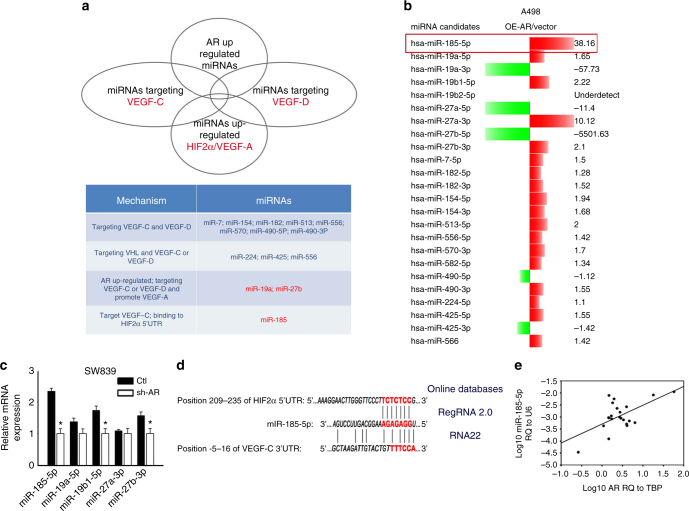
AR regulates HIF2α and VEGF-A vs. VEGF-C via miR-185-5p. **a** The strategy to screen miRNAs (*upper*) and mechanisms of the miRNAs selected (*lower*). **b** *Right* columns and positive values represent increase while *left* columns and negative values represent decrease, and the number equals the fold change. The response to AR overexpression of potential miRNAs was examined in A498 cells. **c** qPCR to screen miRNAs regulated by AR-knockdown in SW839 cells. Triplicate experiments were carried out. *p < 0.05 was considered statistically significant (*t*-test). **d** Sequence alignment of miR-185-5p with HIF2α promoter region (predicted by RegRNA2.0) and 3′ UTR of VEGF-C (predicted by RNA22). **e** Clinical association of AR expression with miR-185-5p expression in ccRCC tumors analyzed by linear regression (*p* = 0.003, *r* = 0.600, *n* = 22)

To dissect the mechanism(s) how AR can differentially modulate the HIF2α→VEGF-A signal vs. VEGF-C signal in the VHL-mut ccRCC cells, we focused on modulation of the miRNAs. The miRNAs have been reported to be able to suppress gene expression via targeting the 3′ untranslated region (UTR) of mRNA. In contrast, they can also enhance gene expressions via targeting their 5′ promoter regions^27, 28^. We then searched the online databases (DIANA miRGen, MicroCosm Targets, RNA22, and RegRNA2.0) and public literature to identify the potential miRNAs candidates (listed in Fig. 4a *lower* panel) that could enhance HIF2α/VEGF-A signal yet suppress VEGF-C and VEGF-D signals (Fig. 4a *upper* panel).

Among these miRNA candidates that can respond to AR modulation in A498 cells (Fig. 4b), we found that miR-185-5p, as well as some of the miR-19 family and miR-27 family, were upregulated by AR, suggesting these AR-modulated miRNAs could either suppress VEGF-C expression via binding to its 3′ UTR^29^ or promote HIF2α and VEGF-A expression via binding to the 5′ promoter region^30^.

To further validate these potential miRNA candidates in AR-meditated regulation of VEGF-A and VEGF-C, we then knocked down AR in SW839 cells and found that miR-185-5p decreased more dramatically than others in response to knocking down AR (Fig. 4c), suggesting miR-185-5p could be our best candidate as it was also predicted to bind to both the 3′ UTR of VEGF-C and 5′ promoter of HIF2α (Fig. 4d), based on Graphic map from RNA22^30^ and RegRNA 2.0^31^, respectively. Importantly, a clinical survey of 22 VHL-mut ccRCC tumors also found that the miR-185-5p was positively correlated with the AR expression (Fig. 4e).

**Fig. 5:**
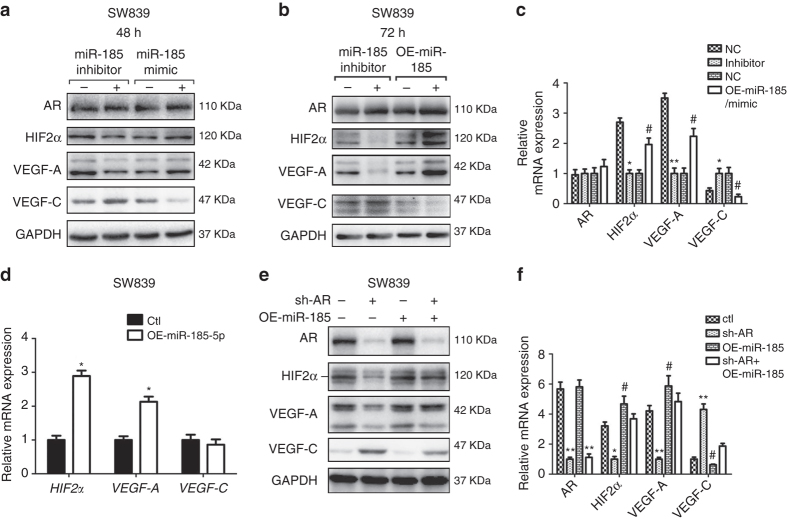
The miR-185-5p regulates expression of VEGF-A and VEGF-C. **a** Immunoblotting of HIF2α, VEGF-A, and VEGF-C with overexpressed or inhibited miR-185-5p in SW839 cells after 48 h treatment with miR-185-5p mimic or inhibitor. **b** Immunoblotting of HIF2α, VEGF-A, and VEGF-C in SW839 cells after 72 h treatment with miR-185-5p overexpressing plasmid or inhibitor. **c** Statistical results of **a**, **b**. **d** qPCR of *HIF2α, VEGF-A*, and *VEGF-C* after overexpressing miR-185-5p in SW839 cells for 48 h. **e** Immunoblotting of HIF2α, VEGF-A, and VEGF-C with/without knockdown of AR and overexpression of miR-185-5p in SW839 cells. **f** Statistical results of **e**. Triplicate experiments were carried out in all experiments. *and ^#^ *p* < 0.05, **p < 0.01 were considered statistically significant by *t*-test for two groups or ANOVA for more than two groups

We then altered the miR-185-5p expression in SW839 cells via either overexpression or knockdown and used immunoblotting analysis to examine their influences on the expression of HIF2α, VEGF-A, and VEGF-C. The results are in agreement with the above predictions showing that overexpression of miR-185-5p or adding functional miR-185-5p mimic promoted expression of HIF2α/VEGF-A and suppressed VEGF-C expression in SW839 cells (Fig. 5a–c). In contrast, treating with miR-185-5p inhibitor suppressed the expression of HIF2α and VEGF-A, and increased the VEGF-C expression in SW839 cells (Fig. 5a–c). The mRNA expression of HIF2α and VEGF-A increased in response to miR-185-5p expression in SW839 cells, while VEGF-C mRNA levels did not show a significant change (Fig. 5d). These results supported the hypothesis that miRNAs can both transcriptionally and post-transcriptionally regulate their target genes.

We then applied an interruption approach to prove that AR might function through promoting miR-185-5p expression to differentially regulate the expression of HIF2α and VEGF-A vs. VEGF-C. As expected, the results revealed that overexpression of miR-185-5p partially reversed the AR-knocked down effects on the expression of HIF2α and VEGF-A vs. VEGF-C in SW839 cells (Fig. 5e, f).

Together, results from Fig. 4a–e and Fig. 5a–f suggest that AR may function through modulation of miR-185-5p to differentially regulate the expression of HIF2α and VEGF-A vs. VEGF-C in ccRCC VHL-mut cells.

### The miR-185-5p regulates expression of VEGF-A vs. VEGF-C

**Fig. 6:**
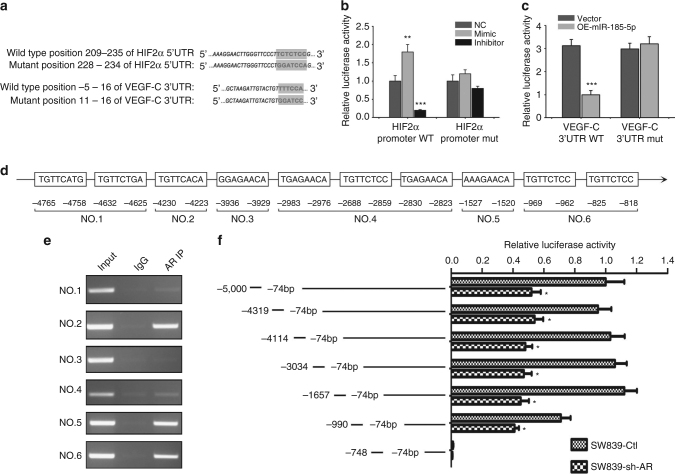
AR regulates expression of miR-185-5p that targets HIF2α and VEGF-C. **a** Mutations introduced at the target sequence of miR-185-5p in HIF2α and 3′ UTR of VEGF-C. **b**, **c** Luciferase reporter assays showing miR-185-5p can target HIF2α promoter (**b**) and VEGF-C 3′ UTR (**c**) in SW839 cells. **d** Bioinformatics analysis of potential AR binding sites on the miR-185-5p promoter using online software ALGGEN PROMO. **e** ChIp assay showing AR can bind to several potential binding sites on the miR-185-5p promoter. **f** Promoter truncation showing that AR binding to region between −1527 to −1520 and −969 to −818 bases of miR-185-5p promoter is important for AR’s regulation of miR-185-5p. Activity of 5K promoter (−5000 to −74) in SW839-control was used as a control. All experiments were carried out in triplicate. *p < 0.05, ***p < 0.001 were considered statistically significant by *t*-test for two groups or ANOVA for more than two groups

We generated pGL3-basic vectors that carry the wild-type and mutant promoter region (−1117 to +257) of HIF2α as well as CMV-driven luciferase followed by the wild-type and mutant 3′ UTR of VEGF-C (Fig. 6a) and measured the luciferase activity in SW839 cells after introduction of these constructs. The results revealed that miR-185-5p could promote the HIF2α promoter activity in SW839 cells (Fig. 6b) and suppress the luciferase expression of VEGF-C 3′ UTR (Fig. 6c), compared to the mutated constructs.

Together, results from Fig. 6a–c suggest that AR-enhanced miR-185-5p could bind to the promoter region of HIF2α to promote the HIF2α and VEGF-A expression and bind to the 3′ UTR of VEGF-C to suppress its expression.

### Mechanism dissection of how AR modulates miR-185-5p expression

We next asked how AR could modulate the expression of miR-185-5p. We applied the miRStart to determine the transcriptional start site of pre-miR-185 and then examined the potential AR binding element (AREs) up to 5000 bases upstream from the transcriptional start site, and results revealed that this promoter region contains 10 potential AR binding elements that may allow AR to bind (Fig. 6d). We applied the chromatin immunoprecipitation assay to verify their capacity for binding to AR, and results indicated that AR could bind to the AR binding elements located on the −4230 to −4223, −1527 to −1520, and −969 to −818 bps in the 5′ promoter region of miR-185-5p in SW839 cells (Fig. 6e). Importantly, luciferase reporter assays driven by a series of truncations of this region further confirmed that AR might function through binding to the region between −1527 and −1520 as well as −969 and −818 bps to modulate the miR-185-5p 5′ promoter activity (Fig. 6f).

Together, results from Fig. 6d–f suggest that AR can enhance the miR-185-5p expression through binding to its 5′ promoter region.

### AR regulation of ccRCC metastatic destinations in vivo

To confirm the validity of the above in vitro data in the in vivo mouse model, we performed the orthotopic implantation of ccRCC SN12-PM6 cells into the left sub-renal capsule of mouse kidney, as early studies indicated that this unique ccRCC cell line could effectively develop tumors and metastases in vivo^32^. We first transduced the SN12-PM6 cells with luciferase and knocked down VHL simultaneously with lentiviral vectors pLV-luci-U6-VHL to generate the stable luciferase expressing VHL-mut cell line (see quantitative PCR/western blot in Supplementary Fig. 2A, B), and in vitro western blot studies confirmed this newly generated VHL-mut cell line has enhanced miR-185-5p and HIF2α expression (Supplementary Fig. 2C, D). Importantly, similar to above studies from the other ccRCC VHL-mut SW839 cells, AR could also differentially regulate VEGF-A and VEGF-C expression likely through upregulation of miR-185-5p (Supplementary Fig. 2C, D). To examine the role of AR and miR-185 in ccRCC progression in vivo, the SN12-PM6 cells with stable transduction of pLV-Luci-U6-VHL were further infected with pLV-puro, pLV-AR, pLV-sh-miRNA-185, or pLV-AR-sh-miR-185.

**Fig. 7:**
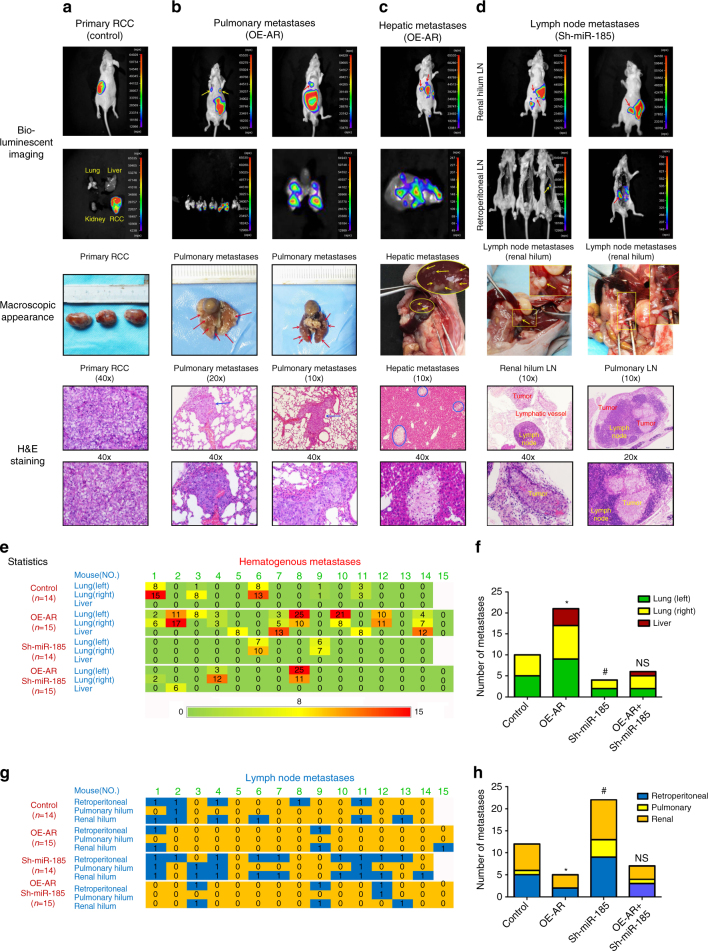
AR regulates differential metastasis in vivo via miR-185-5p. **a**–**d** The animals were killed 12 weeks later for primary RCC and metastases detection by ex vivo bioluminecent imaging (*upper* two panels), gross examination (*middle* panels), and histological staining with haematoxylin and eosin (*H & E*) (*lower* two panels). *Scale bars* in 10×, 20×, and 40× represent 200 μm, 100 μm, and 50 μm, respectively. **a** Primary RCC from control group. Tumor sizes were about 2 cm. **b**, **c** Representative photographs showed pulmonary and hepatic metastases from OE-AR group, macroscopically visible in lung or liver surface metastatic nodules (*arrows* or *circles* indicated) and microscopically visible nodules by H&E staining. **d** Representative photographs of lymph node metastases. Retroperitoneal lymph nodes after resection of all the abdominal organs (*second* panel), and pulmonary or renal hilum lymph nodes were gross examined by anatomical analysis (*third* panel) and confirmed by H&E staining (*lower* two panels). **e** Array diagram showed the hematogenous metastatic status of each mouse in various groups. Whether tumor metastasized or not referred to in/ex vivo bioluminescent or macroscopic imaging and confirmed by H&E staining. Number in each cell represents the visible surface metastatic nodules in left/right lung and liver. *Green* = no metastasis, *red* = > 15 nodules in two layers (with a distance of 3 mm) calculated into total numbers. **f** Quantitation (*χ* ^2^-test) of hematogenous metastases. Each group compared to control group. **g** Array diagram showed the lymphatic metastases of every mouse in various groups (0 = negative, 1 = positive). **h** Quantitation (*χ* ^2^-test) of lymphatic metastases *and # *p* < 0.05 were considered statistically significant. *AR compared to control and #Sh-miR-185-5p compared to control. NS no significance

Sixty male nude mice were randomly divided into four groups for injections with SN16-PM6 control, overexpressed AR (OE-AR), knocked down miR-185-5p (Sh-miR-185), or overexpressed AR together with knocked down miR-185-5p (OE-AR + Sh-miR-185) cell xenografts. The tumors were grown for 12 weeks, with bioluminescent imaging monitoring of both primary and metastatic tumors at 4, 8, 10, and 12 weeks (Fig. 7a–d, *first* panel). We also performed the ex vivo bioluminescent imaging immediately after mice were killed to monitor the lung, liver, and retroperitoneal lymph node metastases (Fig. 7a–d, *second* panel). Anatomic studies were carried out to examine the macroscopic appearance of primary tumors and the metastases (Fig. 7a–d, *third* panel). In addition, histological stainings were performed to confirm the tumor type (Fig. 7a–d, *fourth* and *fifth* panel).

As shown in Fig. 7b–d, ccRCCs cells prefer to metastasize to lung and liver through hematogenous approach and to renal hilum, pulmonary hilum, or retroperitoneal lymph nodes through the lymphatic approach. Hematogenous metastatic capacity was evaluated as number of metastases in left/right lung and liver, while lymphatic metastatic capacity was evaluated as metastases in renal hilum, pulmonary hilum, or retroperitoneal lymph nodes.

**Fig. 8:**
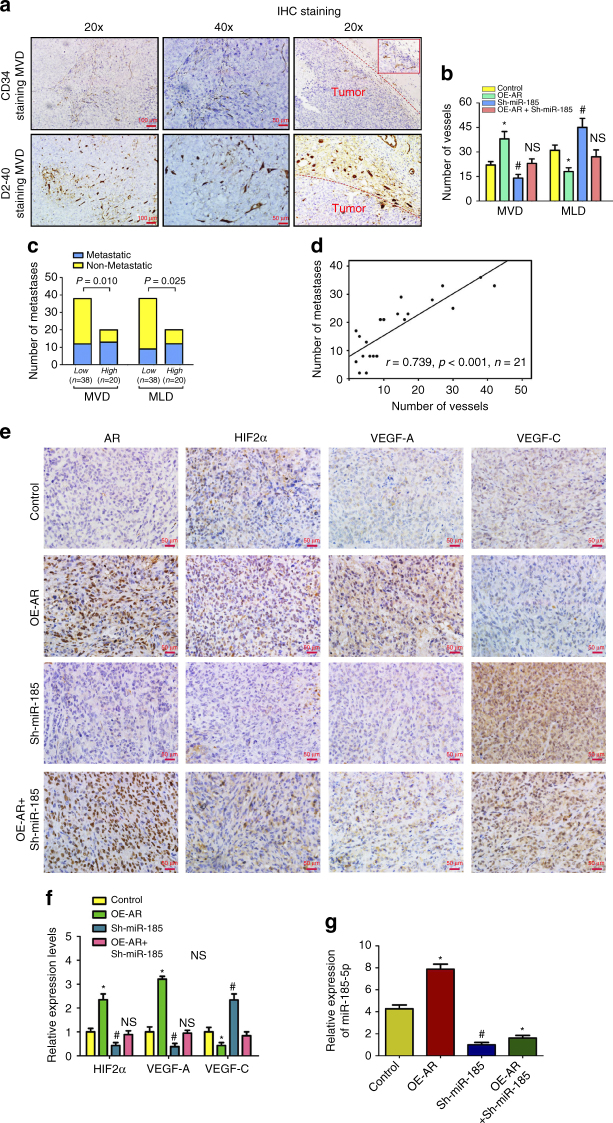
AR influences MVD and MLD by regulating VEGF-A and VEGF-C. After the mice were killed, the primary tumor tissues were fixed with 4% formaldehyde, imbedded in paraffin and processed by immunohistochemistry. **a** Representatives images of IHC staining of CD34 for microvessel density (MVD) and D2-40 for microvessel lymphatic density (MLD) inside or surrounding primary tumors, respectively. *Dashed lines* mark the borders between tumor nodules and surrounding tissues. *Scale bars* in 20× and 40× represent 100 and 50 μm, respectively. **b** Quantitation of angiogenic and lymphangiogenic vessels (MVD vs. MLD) in the various groups (four mice/group). Mean ± SEM of five random views was calculated for every slide and group. **c** Metastatic possibility was stratified by low-level (~2/3, *N* = 38) or high-level (~1/3, *n* = 20) of MVD/MLD (*χ* ^2^-test). **d** Number of metastatic nodules in lung and/or liver was positively correlated with MVD (linear correlation, *r* = 0.739, *p* < 0.001, *n* = 21). **e** Representative picture of IHC staining of AR, HIF2α, VEGF-A, and VEGF-C in the primary RCC. Overexpression of AR increased VEGF-A expression while suppressed VEGF-C expression, but knockdown of miR-185-5p could reverse the results of AR. *Scale bars*, 50 μm. **f** Quantitation of HIF2α, VEGF-A, and VEGF-C expressions detected by immunohistochemistry using Image-Pro Plus software (four mice in every group). **g** Real-time PCR assays for miR-185-5p expression in various groups. Total RNAs were subjected to real-time PCR assays and the relative expressions were plotted as distribution plots. The experiments were carried out in triplicate. *and #*p* < 0.05 were considered statistically significant. *AR compared to control and ^#^Sh-miR-185-5p compared to control. NS no significance (*t*-test for two groups or ANOVA for more than two groups)

The results from these in vivo mouse studies indicated that AR expression could enhance lung and liver metastases (Fig. 7e, f), while suppress peritoneal LM (Fig. 7g, h). However, knocking down miR-185-5p could decrease the metastatic capacity to lung and liver (Fig. 7e, f), while promote the lymph nodes metastases (Fig. 7g, h). Importantly, knocking down miR-185-5p could partially reverse the AR effect on ccRCC cells (Fig. 7e–h). IHC staining of MVD by CD34 and MLD by D2-40 further supported the conclusion showing that overexpression of AR resulted in increased MVD and a decreased MLD while knocking down miR-185-5p decreased MVD, but increased MLD (Fig. 8a, b). Furthermore, knocking down miR-185-5p could also partially reverse the effect of AR (Fig. 8a, b).

We next determined the relationship between vessels density with the metastatic potential, showing that high MVD was correlated with an increasing possibility of hematogenous metastasis and high MLD was correlated with an increasing possibility of lymphatic metastasis (Fig. 8c). In addition, the numbers of blood vessels were positively correlated with the numbers of nodules in lung and liver (Fig. 8d). In Fig. 8e, f, IHC staining of primary RCC tumors in this model showed that compared to controls (*upper* panels) overexpression of AR (*middle* panels) increased HIF2α/VEGF-A expression while suppressing VEGF-C expression. However, knocking down miR-185-5p could reverse the impact of AR (Fig. 8e *bottom* two sets of panels, and Fig. 8f). Moreover, overexpression of AR also upregulated miR-185-5p in vivo (Fig. 8g).

**Fig. 9:**
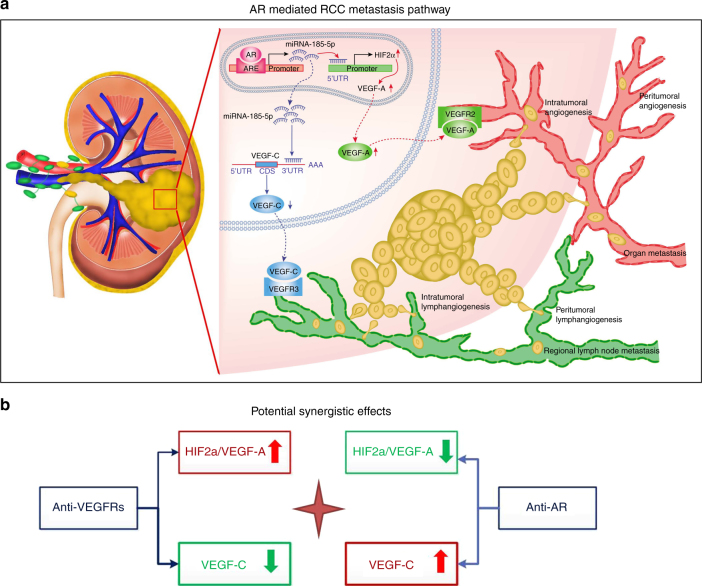
Schematic illustration of AR’s role in RCC metastasis and therapy. **a** A schematic illustration of the proposed model that AR increases hematogenous metastasis yet decreases lymphatic metastasis of RCC. AR increases miR-185-5p by targeting its promoter. The miR-185-5p upregulates HIF2α also by targeting the promoter and results in elevation of VEGF-A. However, miR-185-5p downregulates VEGF-C by targeting the 3′ UTR. **b** A schematic illustration of potential targeted therapeutic strategy of combining anti-VEGFR with anti-AR

Together, these in vivo data were consistent with the conclusion we obtained in the clinical survey and RCC cell lines study in vitro. As summarized in Fig. 9a cartoon, AR increases hematogenous metastasis yet decreases lymphatic metastasis of RCC through differential regulation of the VEGF-A vs. VEGF-C via miR-185-5p.

## Discussion

The ccRCC tumor is highly vascularized with a rich lymphatic return and frequent metastases. During hematogenous or lymphatic metastasis, tumor cells invade the surrounding tissue and enter the hematogenous/lymphatic stream. The surviving cells eventually arrest in the circulation, extravasate into a tissue, and colonize in a new location^23^. Invasion into the hematogenous/lymphatic stream is the first step for ccRCC metastasis. In this study, we proposed that AR plays a dual role in ccRCC metastasis through differential regulation of VEGF-A vs. VEGF-C expression, which affects angiogenesis or lymphangiogenesis and ultimately determines the metastatic sites.

It is unusual that one transcriptional factor, in this case AR, could both promote and suppress site-specific ccRCC metastasis. However, an early report found in the spontaneous metastases of B16 melanoma, host Par2 could also play dual roles that limits the local cancer progression in one area, yet enhances distant metastatic spread^32^. It has been reported that AR expression is positively associated with VEGF-A^33, 34^, while negatively associated with VEGF-C^35^, during prostate cancer progression, yet differential metastatic locations of prostate cancer have not been associated with the opposite regulation of VEGF-A and VEGF-C by AR. We extended those studies and demonstrated the biological consequences of such a differential regulation of VEGF-A and VEGF-C in ccRCC. Moreover, the role of AR in regulating ccRCC metastatic destinations is substantiated in the human clinical population as well as in vivo mouse model studies. Our findings also shed a light on the previous report that AR expression is lower in a panel of ccRCC cell lines and a limited number of clinical samples^9^. It was clear that those samples were enriched with lymph node metastases, thus nicely complementing our current studies.

Mechanism dissection of the differential regulation of VEGF-A and VEGF-C also led us to identify miR-185-5p that likely mediates this effect through its ability to target the 3′ UTR of VEGF-C and to bind to the HIF2α promoter to enhance HIF2α transcription, thus its target gene VEGF-A (Fig. 9a). Whether miR-185-5p is the sole mechanism that mediates the AR’s effect to differentially regulate VEGF-A and VEGF-C is not clear. An earlier report indicated that AR could regulate miR-145 to upregulate HIF2α expression in RCC^36^, thus it is likely that AR may regulate ccRCC metastatic destinations through a combination of various mechanisms.

The ccRCC resists chemotherapy and radiotherapy with a limited therapeutic duration of the anti-angiogenesis targeted therapy (6–15 months^36^). A new and better therapy with clear mechanistic foundations to suppress RCC metastasis is therefore needed to extend patients survival. Our finding that AR can differentially regulate VEGF-A and VEGF-C through the miR-185-5p expression may explain the clinical phenomenon why patients with higher AR-positive ccRCC may have pulmonary metastasis vs. those patients with lower AR-positive ccRCC may have preferential invasion into the lymph nodes. Importantly, these findings suggest that anti-AR therapy may be biased in favor of ccRCC patients with a propensity for lung metastasis and combining anti-AR and anti-VEGF-C therapy should be better to treat this metastatic ccRCC.

Current targeted therapies for metastatic ccRCC, such as Sunitinib (or Sorafenib), the broad spectrum tyrosine kinase inhibitors, can extend RCC patients survival, yet have a therapeutic durability of only 6–15 months^36^. These last-line medications can target both VEGF-A and VEGF-C via suppression of VEGF receptors^37^, and increased HIF1α and HIF2α signals might be linked to the development of resistance during Sunitinib therapy^38, 39^. Thus, suppressing HIF2α and VEGF-A signaling through independent means might extend the efficacy of anti-angiogenesis therapy. Importantly, our findings that AR could enhance HIF2α and VEGF-A signaling, and anti-AR therapy with ASC-J9 suppressed ccRCC progression^40^, suggest that anti-AR therapy may represent a potential novel therapy to overcome the Sunitinib resistance to suppress ccRCC progression. In return, Sunitinib therapy may also be able to overcome the undesirable side effects of anti-AR-enhanced lymphatic metastasis via promoting VEGF-C signals. Therefore, a novel therapy with the combination of Sunitinib and anti-AR therapy could be developed to compensate for and cover each therapy’s unwanted side effects to better suppress RCC progression (summarized in Fig. 9b).

## Methods

### Tissue samples

The epidemiological survey of 3989 cases of RCC patients (from January 2006 to December 2014) was performed on the clinical database of Chinese PLA General Hospital. A total of 119 specimens of ccRCC for real-time PCR or 89 cases of ccRCC for immunohistochemistry staining were randomly extracted from the patient population stratified by metastatic status. All patients provided signed informed consent for the use of their tissues for scientific research. The current study was approved by the Institutional Review Board. All ccRCC cases were confirmed by a senior pathologist (Department of Pathology, PLA General Hospital) and staged based on the 2011 Union for International Cancer Control TNM classification of malignant tumors.

### Cell culture and transfection

The human normal cell line 293T and RCC cell lines, ACHN, Caki-1, A498, OSRC-2, 769-P, and 786-O were originally purchased from American Type Culture Collection (ATCC, Manassas, VA). The SW839 cell line was purchased from Cell Resource Center for Biomedical Research, Tohoku University. The SN12-PM6 cell line was kindly provided by Dr. X.P. Zhang from Union Hospital in Wuhan, China. All RCC cells were cultured in DMEM supplemented with 10% FBS in the humidified 5% CO_2_ environment at 37 °C. The vascular endothelium HUVEC-C cell line was acquired from ATCC and maintained in media containing 10 ng/ml VEGF165 (PeproTech, USA). HDLMVEC cells were purchased from Cell Applications, Inc. (San Diego, CA) and maintained with endothelial cell growth medium in attachment factor solution pre-coated flasks. Cells were authenticated by STR typing and tested to have no mycoplasma contamination before experiments.

To generate AR overexpressing or AR knocked down stable cell populations, SN12-PM6, A498, and SW839 cells were infected with lentiviral vectors, pWPI-AR/pWPI-Vec or pLKO1-sh-AR/pLKO1-scr or pLV-sh-AR/pLV-scr. To overexpress miR-185-5p, the cells were infected with lentiviral vector pLVTHM-miR-185-5p. To knock down miR-185-5p, the cells were infected with lentiviral vector pLVTHM-sh-miRNA-185 that overexpressed the complementary sequence of miR-185-5p as “miRNA scavengers”^41^. The lentiviruses were produced in 293T cells with the psAX2 packaging plasmid and pMD2G envelope plasmid together with the transfer plasmid. After 48 h transfection, virus supernatants were collected for immediate use and/or frozen at −80 °C for later use.

The transient transfection was performed using the Lipofectamine 3000 (Invitrogen) reverse transfection protocol according to the manufacturer’s instructions. The miR-185-5p mimic, miR-185-5p inhibitor, negative controls, and unspecific All Stars negative control RNA, were from Qiagen and used at the final concentrations of 30 nM.

### RNA extraction and quantitative real-time PCR analysis

For RNA extraction, total RNAs were isolated using Trizol reagent (Invitrogen, Grand Island, NY) and 1 µg of total RNA was subjected to reverse transcription using Superscript III transcriptase (Invitrogen).

The miRNAs were also reversed transcribed from total RNA. Briefly, 1 µg of total RNA was processed for poly A addition by adding two units of polymerase with 1 mM ATP in 1× RT buffer at 37 °C for 20 min in 10 μl volume, and then adding 50 pmol anchor primer to 11 μl, and incubating at 65 °C for 5 min, then 4 °C for 2 min. For the last step of complimentary DNA synthesis, we added 2 µl 5× RT buffer, 2 µl 10 mM dNTP, 1 µl reverse transcriptase to total 20 µl, and incubated at 42 °C for 1 h. Quantitative real-time PCR (qRT-PCR) was conducted using a Bio-Rad CFX96 system with SYBR green to determine the mRNA expression level of a gene of interest or with Tagman probe to determine the miRNA expressions. Expression levels were normalized to the expression of TATA box binding protein TBP RNA, or 5s RNA and/or U6. The primers used for the genes of interest were listed in Supplementary Table 1.

### Western blot analysis

Cells were lysed in RIPA buffer and proteins (30 µg) were separated on 8–10% SDS/PAGE gel and then transferred onto PVDF membranes (Millipore, Billerica, MA). After blocking membranes with non-fat milk solution, they were incubated with appropriate dilutions of specific primary antibodies. For human ccRCC samples and cells, the primary antibodies of the rabbit anti-AR (Santa Cruz, sc–7305), the mouse anti-HIF2α (Abcam, ab8365), rabbit anti-VEGF-A (Abcam, ab46154), rabbit anti-VEGF-C (Abcam, ab135506), and rabbit anti-VEGF-D (GeneTex, GTX100805) were used for blotting at dilutions of 1:2000, 1:500, 1:1000, 1:500, and 1:1000, respectively. They were then incubated with HRP-conjugated secondary antibodies and visualized using the ECL system (Thermo Fisher Scientific, Waltham, MA). The antibodies used were listed in Supplementary Table 2 and the indicated molecular weights in uncropped scans were shown in Supplementary Information.

### Endothelial cell migration and invasion assay

Endothelial cells (HUVEC and HDLMVEC) were co-cultured with RCC cells with indicated treatments (with/without overexpression/knockdown of AR) for 72 h, and then the endothelial cells were harvested and subjected to the migration and invasion capability of endothelial cells (HUVEC and HDLMVEC) as determined by the wound-healing and transwell assays, respectively. For these experiments, the endothelial cells were maintained in mixed CM, media from the co-culture system with fresh media at the ratio of 1:1. Wound-healing assays of HUVEC and HDLMVEC cells co-cultured with A498 and SW839 with altered AR expression were conducted at indicated time points. The cells migrated into the wounds were counted after 24 h. For the invasion assay, before seeding the cells, 100 µl of Matrigel (BD, Inc., Franklin Lakes, NJ) was dissolved in 1.5 ml serum-free DMEM and applied to the upper chambers of transwells with 8 µm-pore-size polycarbonate membrane filters (Corning, Inc., Corning, NY) and then were incubated at 37 °C for 5 h. Endothelial cells were then harvested and seeded with serum-free DMEM into the upper chambers of the transwells at 1 × 10^5^ cells/well, and the bottom chambers contained mixed CM with 10% FBS, and then incubated for 24 h at 37 °C. Following incubation, the invaded cells attached to the lower surface of the membrane were fixed by 4% paraformaldehyde and stained with 1% crystal violet, while the upper surface and non-attached cells were washed and removed, then cell numbers were counted in five randomly chosen microscopic fields (×400) per membrane.

### Tube formation assay

This experiment was performed using the In Vitro Angiogenesis Assay Kit Tube Formation (CULTREX, Gaithersburg, MD) according to the manufacturer’s instructions. Briefly, after co-culturing with RCC cells, endothelial cells were stained by treating with 2 μM Calcein AM for 30 min, then 5 × 10^4^ cells were harvested and seeded to 48-well plates pre-coated with the BME Reduced Growth Factor. After 6–12 h, the fluorescence microscope (485 nm excitation/520 nm emission) was used to visualize tube formation of the endothelial cells.

### Luciferase assay

The 3′ UTR of VEGF-C was constructed into PWPI-LUC- vector (Promega, Madison, WI, USA) and promoter regions of miR-185-5p and HIF-2α were constructed into pGL3-basic vector (Promega). Cells were plated in 24-well plates and the complimentary DNA were transfected using Lipofectamine 3000 (Invitrogen) according to the manufacturer’s instructions. The pRL-TK was used as an internal control. Luciferase activity was measured by Dual-Luciferase Assay reagent (Promega) according to the manufacturer’s manual.

### Chromatin immunoprecipitation assay and promoter analysis

Cell lysates were precleared sequentially with normal rabbit IgG (sc-2027, Santa Cruz Biotechnology) and protein A-agarose. Anti-AR antibody (2.0 µg) was added to the cell lysates and incubated at 4 °C overnight. For the negative control, IgG was used in the reaction. Specific primer sets designed to amplify a target sequence within the human miR-185-5p promoter were listed in the Supplementary Table 3 and PCR products were identified by agarose gel electrophoresis. For the generation of truncated miR-185-5p promoter, regions of indicated lengths were amplified using the primers listed in Supplementary Table 4 from the original 5K promoter and constructed into pGL3-basic vector (Promega). Luciferase assays were applied to detect the promoter activity.

### In vivo metastasis studies

Four-to-six-week-old male BALB/c nude mice were housed and fed under specific pathogen-free conditions. The animal models in this study were approved by the Animal Ethical Committee of the Chinese PLA General Hospital. We first generated stable SN12-PM6 cells with lentiviral vectors of pLV-Luci-U6-VHL and luciferase. This stable cell line was further divided and infected with pLV-puro, pLV-AR, pLV-sh-miRNA-185, or pLV-AR-sh-miR-185. About 60 male nude mice were randomly divided into four groups without blinding for injection of cells with control, overexpression of AR (OE-AR), knockdown of miR-185-5p (Sh-miR-185), and overexpression of AR with simultaneous knockdown of miR-185-5p (OE-AR + Sh-miR-185). The luciferase stable expressing SN12-PM6-sh-VHL cells (at 1 × 10^6^, mixed with Matrigel, 1:1) with various transduction listed above were then injected orthotopically into capsule of left kidney of nude mice (*n* = 15). Two mice died of anesthesia in the control group and in the Sh-miR-185 group. A bioluminecent imaging system (NightOWL II, LB983, Berthold Technologies, Germany) was used to monitor the primary and metastatic lesions following abdominal injection of 150 mg/kg Luciferin at four different time points (4, 8, 10, and 12) weeks. The animals were killed at the end of 12 weeks, metastases were detected by in/ex vivo bioluminecent imaging, gross examination, and histological staining with haematoxylin and eosin (H & E).

### Immunohistochemistry staining

The kidneys with tumors, as well as any metastases, were fixed in 4% neutral buffered paraformaldehyde and embedded in paraffin. For human ccRCC samples, the primary antibodies of the rabbit anti-AR (Santa Cruz), the rabbit anti-HIF2α (Abcam), the rabbit anti-CD34 (Abcam), the mouse anti-D2-40 (Abcam), rabbit anti-VEGF-A (Abcam), and rabbit anti-VEGF-C (Abcam) were used for staining at dilutions of 1:100, 1:1000, 1:100, 1:40, 1:100, and 1:50, respectively. For mouse samples, we used rabbit polyclonal antibody to HIF2α (Abcam) and D2-40 (GeneTex) at dilutions of 1:1000 and 1:40, respectively. The primary antibody was recognized by the biotinylated secondary antibody (Vector), and visualized by VECTASTAIN ABC peroxidase system and peroxidase substrate DAB kit (Vector).

### Statistical analysis

Data were expressed as mean ± SEM from at least three independent experiments. Statistical analyses involved *t*-test or ANOVA with SPSS 17.0 (SPSS Inc., Chicago, IL) after estimating the variance between groups. Linear correlation analyses were performed to determine the correlation between the gene expression levels. The logistic regression model was performed to select the risk factors associated with AR expression. The sample size of human tissues or animals was estimated by preliminary data. *p* < 0.05 was considered statistically significant.

### Data availability

All relevant data are available within the article and Supplementary Files, or available from the authors upon request.

## Electronic supplementary material

Supplementary Information
