# Supplementary material for: Androgen receptor increases hematogenous metastasis yet decreases lymphatic metastasis of renal cell carcinoma
Source: Nat Commun. 2017 Oct 13;8:918. doi: 10.1038/s41467-017-00701-6 (PMC5640635; doi:10.1038/s41467-017-00701-6)
Supplement: Supplementary file 1 — Supplementary Information [file 41467_2017_701_MOESM1_ESM.pdf]

Description of Supplementary Files

File Name: Supplementary Information
Description: Supplementary Figures and Supplementary Tables

Supplementary Table 1. qRT-PCR primers

Target genes	Sense	Antisense
AR	CCAGGGACCATGTTTTGCC	CGAAGACGACAAGATGGACAA
HIF2A	GTCTCTCCACCCCATGTCTC	GGTTCTTCATCCGTTTCCAC
VEGF-A	AGGGCAGAATCATCACGAAGT	GCTGCGCTGATAGACATCCA
VEGF-C	GCAACATAACAGAGAACA	CTCCACTCATTATCAATACTT
VEGF-D	TACCAACACATTCTTCAA	CATACAGATAAGGCTCTC
TBP	CACGAACCACGGCACTGATT	TTTTCTTGCTGCCAGTCTGGAC

Supplementary Table 2. Antibodies used

Antibody	Source	Company (catalog)
AR	Mouse monoclonal antibody	Santa Cruz (sc–7305)
AR	Rabbit polyclonal antibody	Santa Cruz (sc–815)
VHL	Rabbit polyclonal antibody	Cell Signaling(2378S)
HIF2a	Mouse monoclonal antibody	ABcam(ab8365)
HIF2a	Rabbit polyclonal antibody	ABcam(ab109616)
VEGF-A	Rabbit polyclonal antibody	ABcam(ab46154)
VEGF-A	Rabbit polyclonal antibody	Santa Cruz (sc–152)
VEGF-C	Rabbit polyclonal antibody	ABcam(ab135506)
VEGF-C	Rabbit polyclonal antibody	Santa Cruz (sc–25783)
VEGF-C	Rabbit polyclonal antibody	GeneTex(GTX113574)
VEGF-D	Rabbit polyclonal antibody	GeneTex(GTX100805)
CD34	Rabbit monoclonal antibody	ABcam(ab81289)
D2-40	Mouse monoclonal antibody	ABcam(ab77845)
D2-40	Rabbit polyclonal antibody	GeneTex(GTX12025)

Supplementary Table 3. Primers for ChIp assay of miR-185-5p promoter

Position	Sense	Antisense
NO.1	ACAGCTTTCAGCCTTCCTCA	GGACCATGACCACAAAGGAC
NO.2	TGCTGCTCCTACTCCAAGGT	GGACCCAGTTTCTCCATGTG
NO.3	TGAGCCGTCATCTCACTGAC	CTTCCCAGGGTCTGCTATTG
NO.4	TCTCCTCGCTTCCTGCTCTA	TGTGGTCTGTGCCTCAATGT
NO.5	ATGTTGGCCAGGCTAGTGTC	GAGTCAGGGCCCATTCTGTA
NO.6	GCAGGATTGAGGGTCAGAAC	CCATCCTATCCGCAATGTCT

Supplementary Table 4. Primers for truncate assay of miR-185-5p promoter

Position	Sense	Antisense
-5000~ -74bp	TTCCTGGCCCTGGAGCTGTG	TGAGGGAGACTGCTCGGTTT
-4319~ -74bp	TGCTGCTCCTACTCCAAGGT	TGAGGGAGACTGCTCGGTTT
-4114~ -74bp	TGAGCCGTCATCTCACTGAC	TGAGGGAGACTGCTCGGTTT
-3034~ -74bp	TCTCCTCGCTTCCTGCTCTA	TGAGGGAGACTGCTCGGTTT
-1657~ -74bp	ATGTTGGCCAGGCTAGTGTC	TGAGGGAGACTGCTCGGTTT
-990~ -74bp	GCAGGATTGAGGGTCAGAAC	TGAGGGAGACTGCTCGGTTT
-748~ -74bp	GCAGACATTGCGGATAGGATG	TGAGGGAGACTGCTCGGTTT

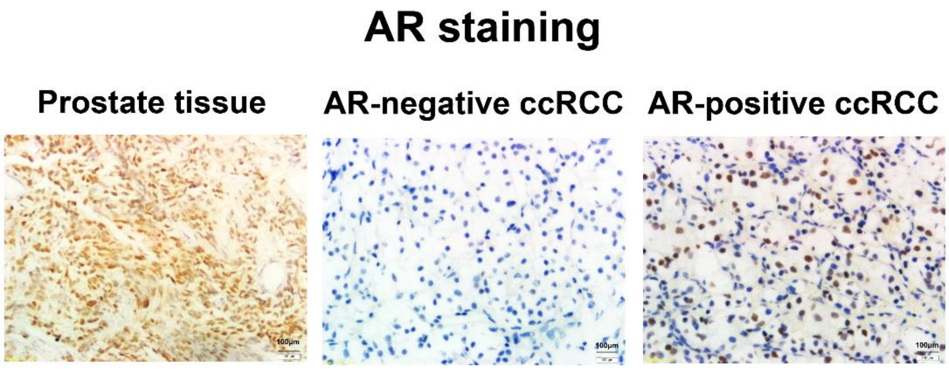

Supplementary Figure 1. AR staining in various ccRCC tumors, prostate tissue AR staining served as a positive control. AR expression was mainly located in the nucleus.

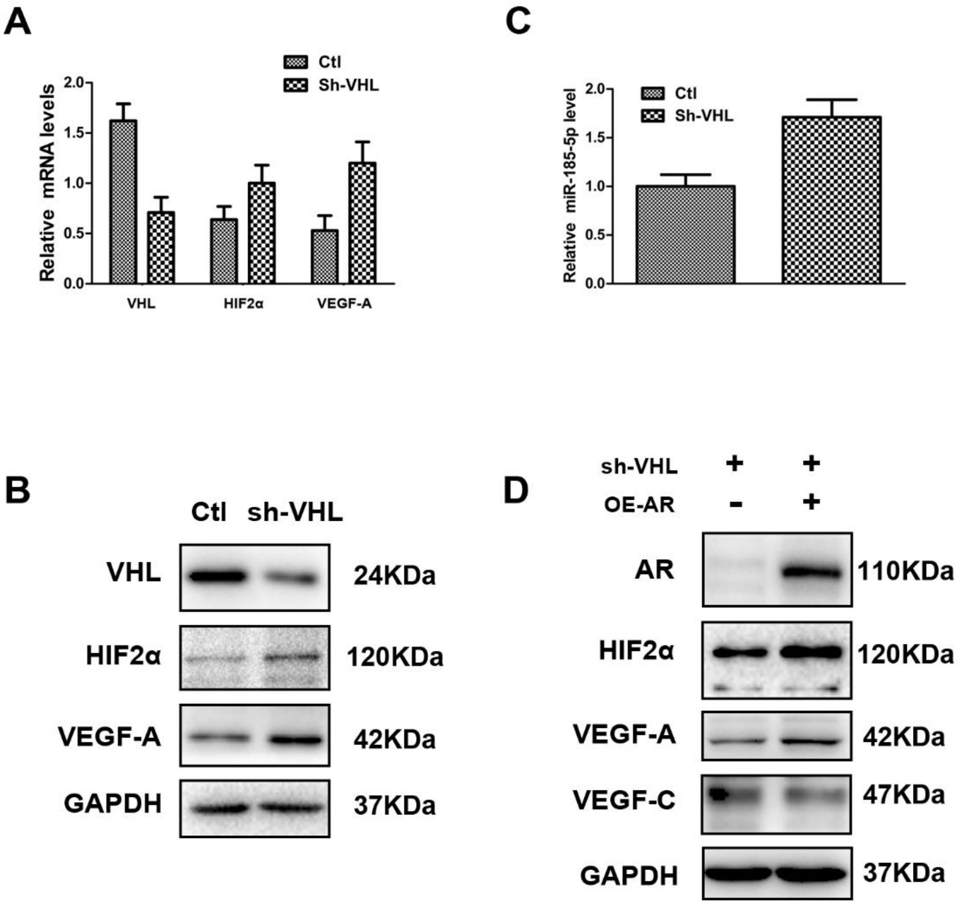

Supplementary Figure 2. Knocking down VHL in SN12-PM6 cells mimicks VHL deficiency in RCC cells. (A). qPCR showing knocking down VHL increased HIF2α and VEGF-A mRNA levels in SN12-PM6 cells; (B). Western blot showing knocking down VHL increased HIF2α and VEGF-A mRNA levels in SN12-PM6 cells; (C). Overexpression of AR increased miR-185-5p in VHL-knocked down SN12-PM6 cells; (D). AR induced VEGF-A yet reduced VEGF-C in VHL-knocked down SN12-PM6 cells.

Protein ladder

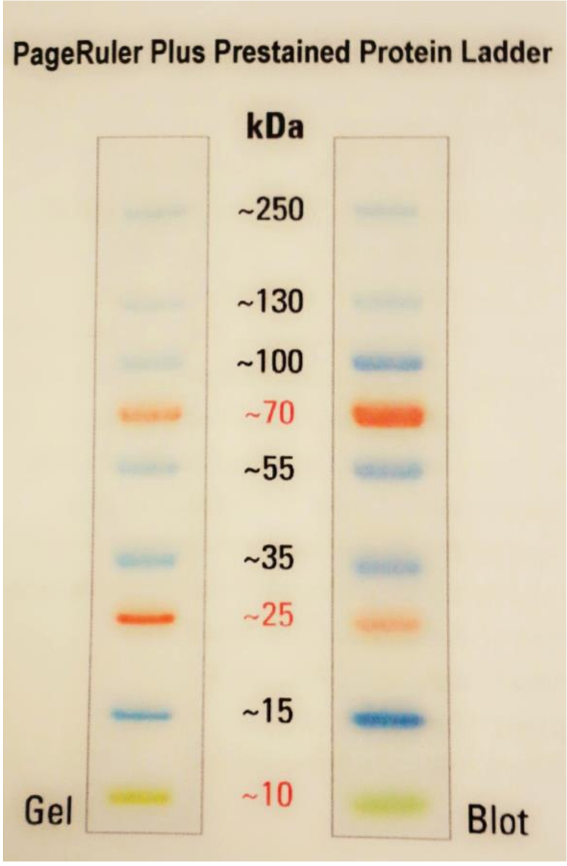

Ponceau S staining （10% Gel）

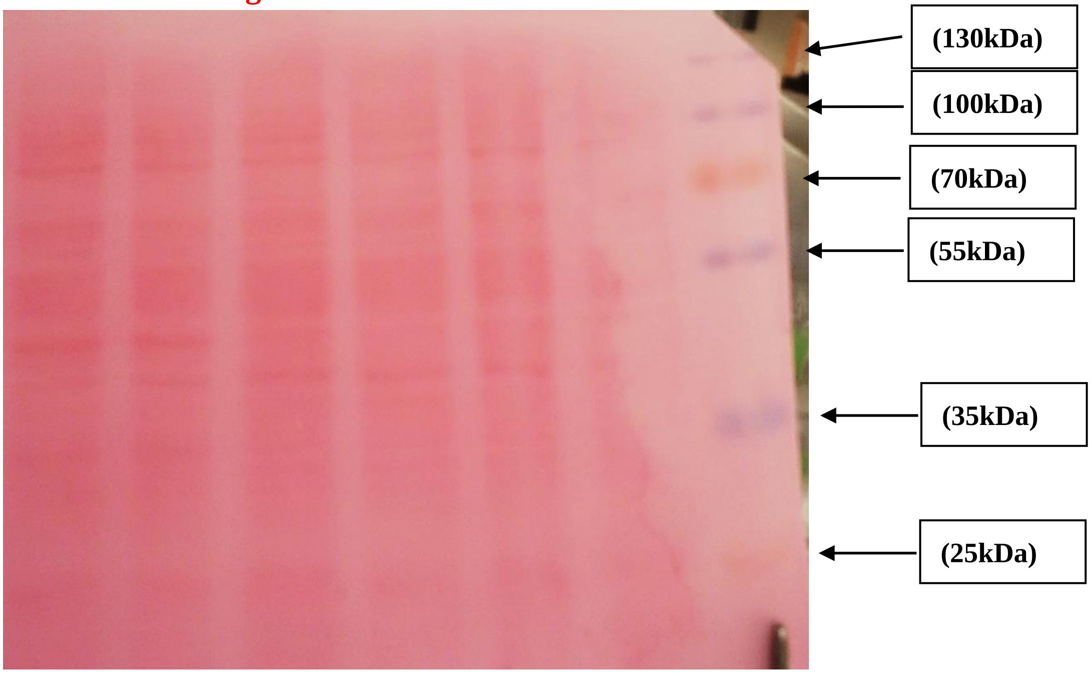

Supplementary Figure 3. Uncropped scans of the western blots of AR, HIF2α, VEGF-A, VEGF-C, VEGF-D and GAPDH (Figure 2C).

AR (110kDa)

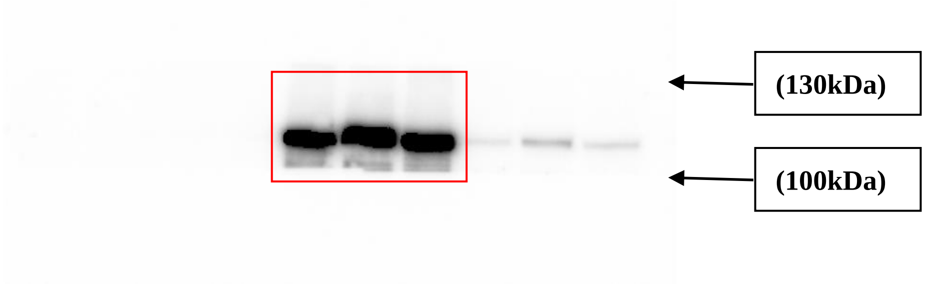

HIF2α (120kDa)

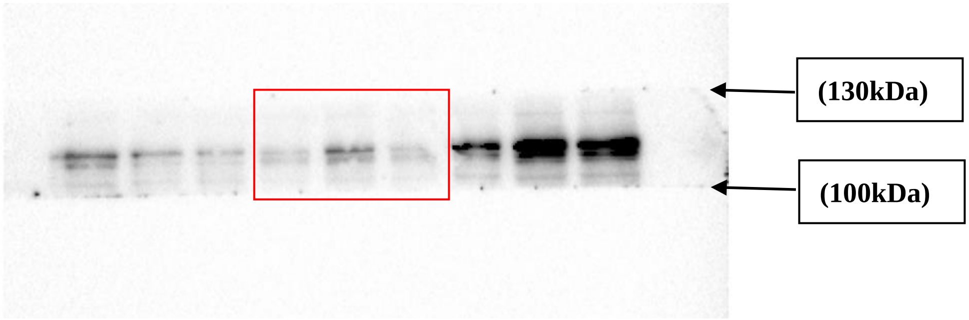

VEGF-A (42kDa)

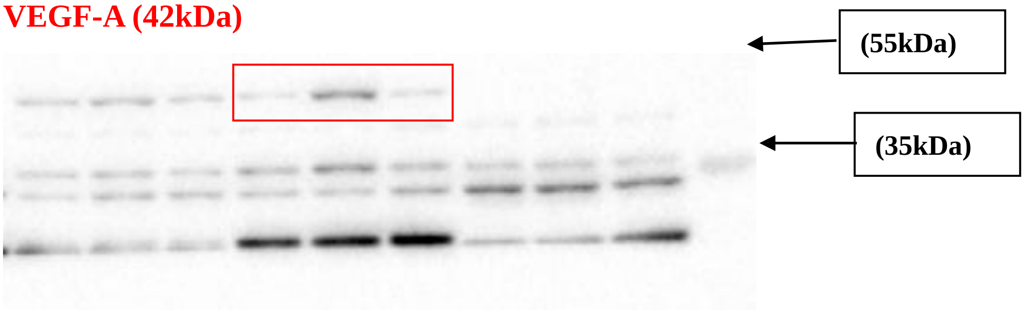

VEGF-C (47kDa)

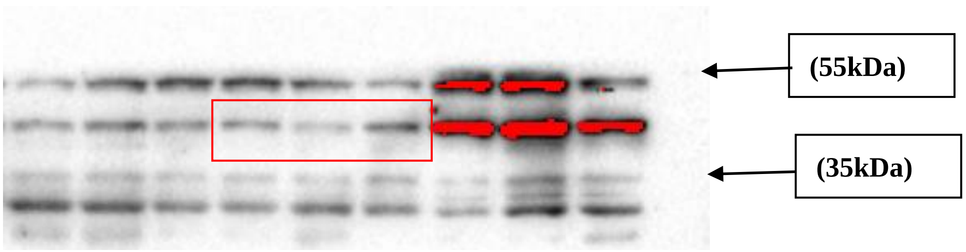

Supplementary Figure 3 (Continued). Uncropped scans of the western blots of AR, HIF2α, VEGF-A, VEGF-C, VEGF-D and GAPDH (Figure 2C).

VEGF-D (40kDa)

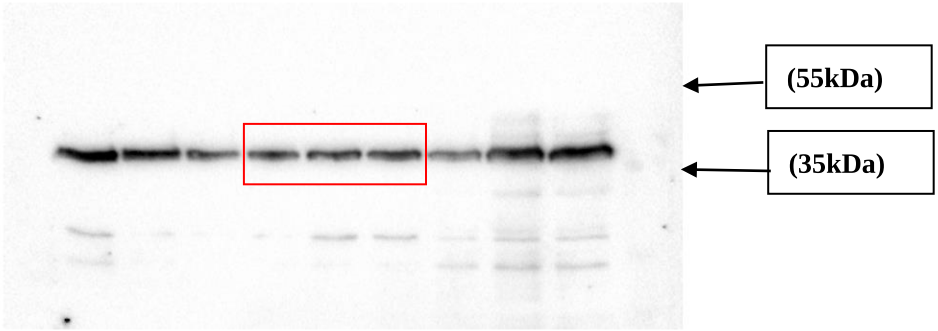

GAPDH (37kDa)

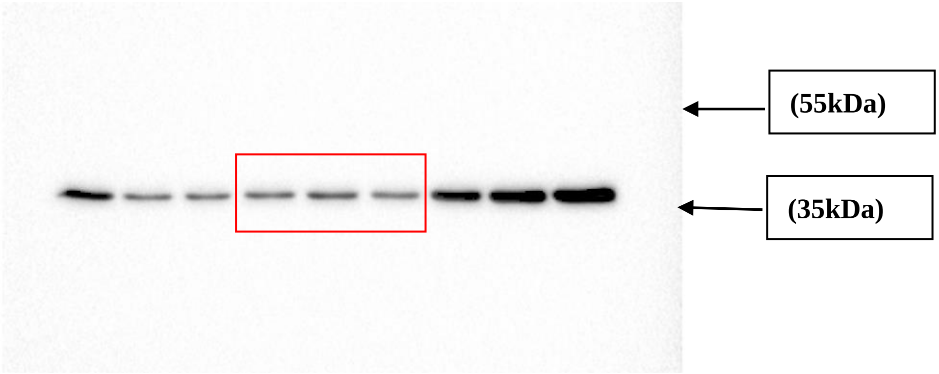

Supplementary Figure 3 (Continued). Uncropped scans of the western blots of AR, HIF2α, VEGF-A, VEGF-C, VEGF-D and GAPDH (Figure 2C).
